# Outcomes of genetic testing for Usher syndrome in a diverse population cohort from South Florida

**DOI:** 10.1186/s40246-025-00775-0

**Published:** 2025-06-18

**Authors:** Zachary J. Cromar, Ryan Chen, Tamara Juvier Riesgo, Denise Yan, Lindsay Dawn Verma, Zhengyi Chen, Susan H. Blanton, Byron L. Lam, Xue Zhong Liu

**Affiliations:** 1 Department of Human Genetics, Dr. John T. Macdonald Foundation, University of Miami Miller School of Medicine, Miami, USA; 2 Bascom Palmer Eye Institute, University of Miami Miller School of Medicine, Miami, USA; 3 Department of Otolaryngology, University of Miami Miller School of Medicine, Miami, USA; 4 Eaton-Peabody Laboratory, Massachusetts Eye and Ear Infirmary, Department of Otolaryngology- Head and Neck Surgery, Harvard Medical School, Boston, USA; 5 John P. Hussman Institute for Human Genomics, University of Miami Miller School of Medicine, Miami, USA

**Keywords:** Usher syndrome, Deafness, Blindness, Minorities, Retinitis pigmentosa

## Abstract

**Background:**

Usher syndrome (USH) is the leading genetic cause of congenital deaf blindness worldwide. USH is an autosomal recessive disorder clinically characterized by partial or complete congenital sensorineural hearing loss followed by progressive vision loss due to retinitis pigmentosa. There are three main subtypes (USH1, USH2, USH3) with different genetic causes categorized by age of symptom onset and severity. Understanding the genetic epidemiology of USH can help identify novel mutations and facilitate definitive diagnosis and treatment. This retrospective study characterizes the mutation spectrum of USH in an ethnically diverse South Florida population.

**Results:**

Of the 148 patients assessed for this study, 67 were male and 81 were female. In this population, one identified as American Indian or Alaska Native, 6 identified as Asian (A), eight identified as Black or African American (AA), eight identified as More than One Race, 26 were identified as Unknown or Not Reported, and 99 were identified as white. In addition, 42 identified as Hispanic or Latino, 87 identified as Non-Hispanic or Latino, and 19 were identified as Unknown or Not Reported; all individuals identifying as Hispanic or Latino were either White or Unknown. One American Indian or Alaska Native patient, two Asian patients, two Black or African American Patients, and 15 white patients had inconclusive molecular testing results. In our population, White Non-Hispanics were more likely to receive a conclusive molecular diagnosis for their hearing loss.

**Conclusions:**

This is the first genetic characterization of an ethnically diverse South Florida population with USH, which can help direct patient diagnosis and medical care. As clinical trials for treatment increases, molecular testing in all individuals is imperative.

**Supplementary Information:**

The online version contains supplementary material available at 10.1186/s40246-025-00775-0.

## Background

Congenital or early onset bilateral sensorineural hearing loss combined with retinitis pigmentosa (RP) is a rare disorder with very significant impacts on quality of life. Usher syndrome (USH) is the leading cause of congenital deaf-blindness, accounting for 50% of all cases and affecting ~ 400,000 individuals worldwide [1, 2].

USH, an autosomal recessive disorder, is clinically characterized by partial or complete congenital sensorineural hearing loss (SNHL) followed by progressive vision loss due to RP. There are three main subtypes categorized by age of symptom onset and severity. Type 1 (USH1) is characterized by early onset profound congenital SNHL and vestibular dysfunction and RP in the first decade of life and is the most common type [2]. Type 2 (USH2) manifests during late adolescence or early adulthood with RP and moderate SNHL but preserved vestibular function. Type 3 (USH3) is also characterized with early onset RP, but typically exhibits progressive SNHL and vestibular dysfunction. However, some cases do not meet the aforementioned categories and could be categorized as atypical USH syndrome, because of uncharacteristic audiovestibular or retinal findings [2, 3]. Current care of USH involves managing and monitoring the RP, SNHL, and vestibular function. There are currently no effective treatments to restore vision, but cataract surgery can help improve visual acuity in the 50% of patients who also develop cataracts [4]. Hearing loss is treated with hearing aids or cochlear implantation, and vestibular dysfunction requires rehabilitation [2]. Several clinical trials are also underway investigating the use of antioxidant and targeted gene therapies for the slowing of USH progression [5, 6].

USH has 16 known causative genes, all of which are inherited in an autosomal recessive fashion [7, 8]. However, most USH-causing variants occur in two genes: *MYO7A* and *USH2A* [9, 10, 11]. The proteins encoded by these USH genes interact with one another in an interactome; the dysfunction or absence of either of these proteins leads to retinal and cochlear sensorineural decay [2]. Expanding the genetic epidemiology of USH can help identify novel mutations and facilitate definitive diagnosis and treatment.

Previous studies have shown variety in the spectrum of causative USH genes in both racially homogeneous and heterogeneous populations [12, 13, 14]. Yet, a comprehensive investigation of USH variants has not been conducted in diverse Hispanic or black populations. In the South Florida region, 45.9% of the population identifies as Hispanic, 19.7% identifies as Black or African American (Non-Hispanic), and 41.4% of residents identify as foreign-born. Many of these foreign-born residents share common birthplaces in Cuba, Haiti, and Colombia [15]. This retrospective study seeks to characterize the genetic mutation spectrum of USH in an ethnically diverse South Florida population.

## Methods

### Statement of ethics

This study was performed following the guidelines of the Declaration of Helsinki and was completed under University of Miami Institutional Review Board #20,010,415 as part of a chart review.

### Patient selection

In total, 148 patients were selected for this study from a comprehensive database of patients with USH seen within the University of Miami Health system at Bascom Palmer Eye Institute and the Department of Otolaryngology. Their diagnosis was classified as USH1, USH2, USH3, or atypical USH based on their clinical presentation.

### Data extraction

Demographic data was collected from the electronic medical record via the University of Miami data broker service. Extracted fields included race, ethnicity, sex, and age. Race and ethnicity were self-reported by the participants out of the following options: White, Hispanic; White, Non-Hispanic; Black or African American, American Indian or Alaska Native, and Asian.

We manually located and extracted patient genetic reports that confirmed a molecular diagnosis for USH. Genetic testing was performed by the following laboratories: Baylor College of Medicine Medical Genetics Laboratories (Houston, TX), Blueprint Genetics (Seattle, WA), OHSU Casey Eye Institute (Portland. OR), eyeGENE (Bethesda, MD), GeneDX (Gaithersburg, MD), Invitae (San Francisco, CA), John and Marcia Carver Nonprofit Genetic Testing Laboratory (Iowa City, IA), Massachusetts Eye and Ear Infirmary (Boston, MA), Molecular Vision Laboratory (Hillsboro, OR), and Prevention Genetics (Marshfield, WI). Molecular reports from these laboratories indicated the pathogenicity of each variant identified. Although not all test panels included all of the following genes, they each tested for genes that are known to cause USH including: *MYO7A*, *CDH23*, *PCDH15*, *USH1C* (Harmonin), *USH1G* (SANS), *CIB2*, *USH2A* (Usherin), *ADGRV1*, *WHRN*, *PDZD7*, *CLRN1* and *HARS1* [7]. Genetic variant data collected from these reports included gene affected, cDNA alteration, protein sequence alteration, zygosity, and variant classification for each patient allele.

Molecular diagnoses from genetic testing and clinical diagnoses were confirmed using NIH ClinVar ((http://www.ncbi.nlm.nih.gov/clinvar/), dbSNP, and gnomAD databases (https://gnomad.broadinstitute.org/) for known pathologic variants [16, 17, 18, 19].

### Data analysis

All statistical analyses were performed using R (version 4.4.0). Comparisons between patients with and without genetic testing across groups were performed using Fisher’s exact test to account for small sample size [20]. Values are presented as counts. Fisher’s exact test was performed between patients with and without testing and patients with and without conclusive diagnoses. *P < 0.05.

## Results

**Fig. 1:**
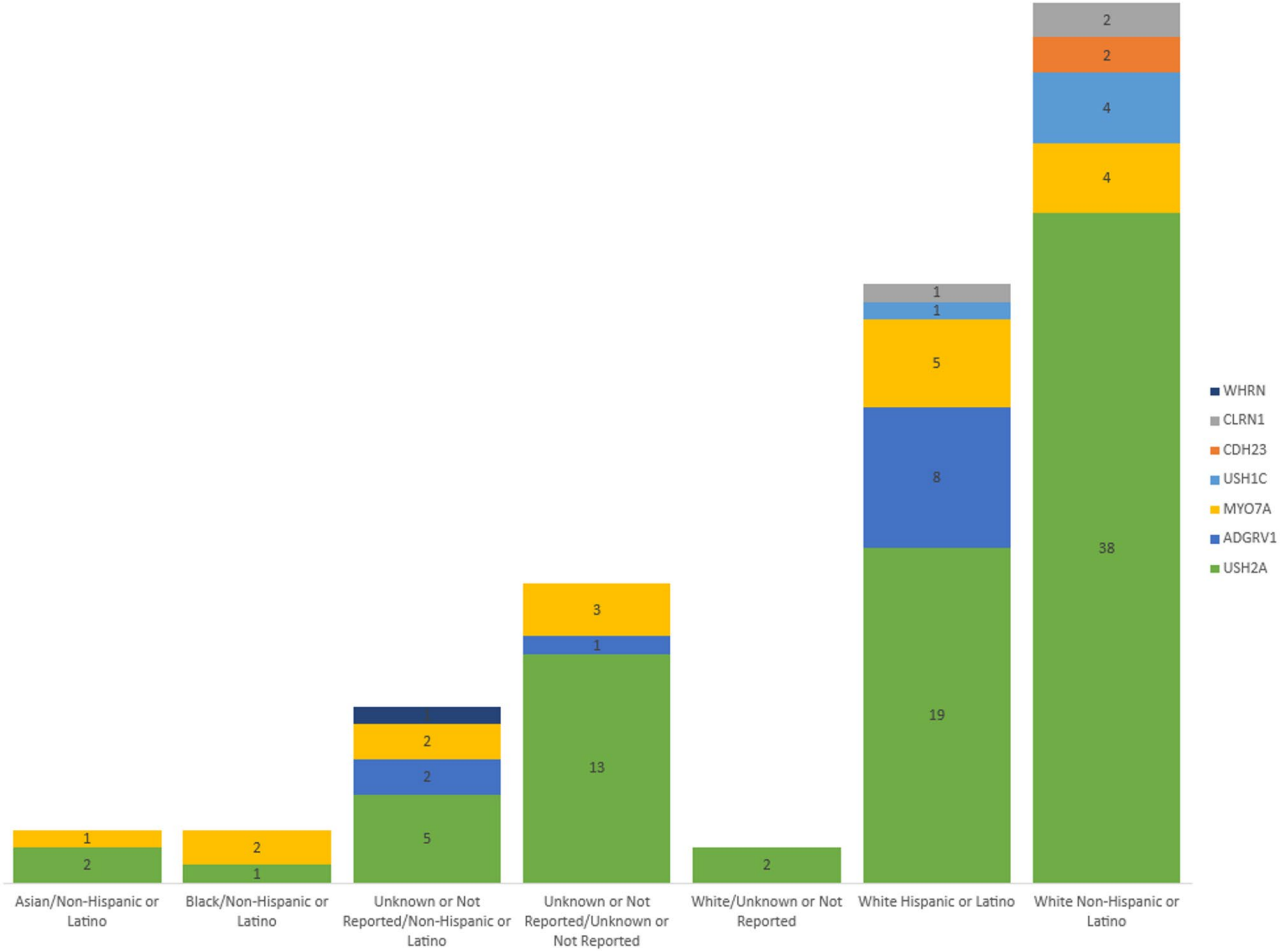
Distribution of variant genes by race/ethnicity. The frequency of genes containing a variant by race-ethnicity categories are demonstrated. *USH2A*, in green, is the most common affected gene and is found in each of the race-ethnicity groups. Variants in *WHRN*, *CLRN1*, *CDH23*, *USH1C*, *MYO7A*, and *ADGRV1*, were also identified

**Fig. 2:**
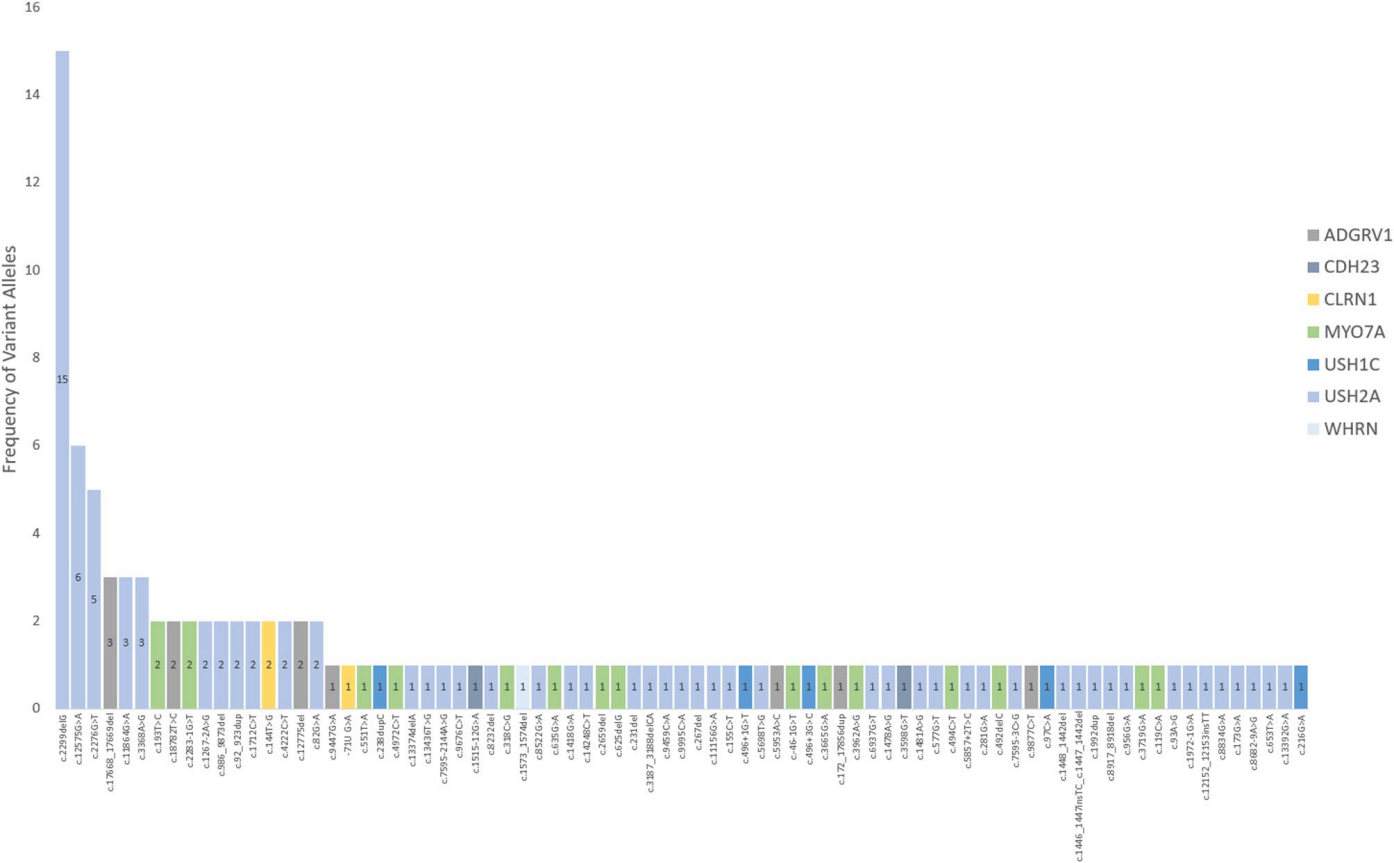
Distribution of variant alleles by gene. The frequency of each variant by gene is shown. Variant c.2299delG in *USH2A* is the most common variant, followed by c.12575G > A in *USH2A*

Of the 148 patients whose charts we reviewed for this study, only 70 had molecular testing results included in their charts. Overall, 71 were male and 83 were female (Table 1). In the total population with available demographic information, one identified as American Indian or Alaska Native, six identified as Asian (A), eight identified as Black or African American (AA), eight identified as More than One Race, 26 identified as Unknown or Not Reported, and 99 identified as white. Forty-two identified as Hispanic or Latino, 87 identified as Non-Hispanic or Latino, and 19 identified as Unknown or Not Reported. All individuals identifying as Hispanic or Latino also identified as either White (40), Unknown [1] or More than One race [1]. The distribution of variant genes by race-ethnicity is provided in Fig. 1. The distribution of variant alleles by gene can be seen in Fig. 2. The distribution of pathogenic variants by race-ethnicity and USH subtypes is provided in Supplemental Table 1. While testing rates were similar across all race/ethnic groups (*p* = 0.14), individuals reporting as White/Non-Hispanic were more likely to receive a molecular diagnosis than other race/ethnic groups (XX vs. XX (*p* = 0.0214).

**Table 1:** Patients with Usher syndrome with and without genetic test results

	Tested (*N* = 70)		Conclusively Diagnosed (*N* = 47)		Inconclusively Diagnosed (*N* = 23)		Not Tested (*N* = 84)	
Male	29	41.4%	23	48.9%	6	26.1%	42	50.0%
Female	41	58.6%	24	51.1%	17	73.9%	42	50.0%
White: Non-Hispanic or Latino	26	37.1%	21	44.7%	5	21.7%	31	36.9%
White: Hispanic or Latino	21	30.0%	12	25.5%	9	39.1%	20	23.8%
Unknown or Not Reported: Unknown or Not Reported	10	14.3%	7	14.9%	3	13.0%	12	14.3%
Unknown or Not Reported: Non-Hispanic or Latino	6	8.6%	6	12.8%	0	0.0%	3	3.6%
Asian: Non-Hispanic or Latino	3	4.3%	1	2.1%	2	8.7%	3	3.6%
Black or African American: Non-Hispanic or Latino	2	2.9%	0	0.0%	2	8.7%	5	6.0%
American Indian or Alaska Native: Non-Hispanic or Latino	1	1.4%	0	0.0%	1	4.3%	0	0.0%
White: Unknown or Not Reported	1	1.4%	0	0.0%	1	4.3%	1	1.2%
More Than One Race: Hispanic or Latino	0	0.0%	0	0.0%	0	0.0%	1	1.2%
More Than One Race: Non-Hispanic or Latino	0	0.0%	0	0.0%	0	0.0%	7	8.3%
Unknown or Not Reported: Hispanic or Latino	0	0.0%	0	0.0%	0	0.0%	1	1.2%

Among patients with genetic test results one American Indian or Alaska Native patient, two Asian patients, two AA patients, and 15 white patients had inconclusive diagnostic results. 122 variants were found, with 72 classified as pathogenic, 5 as likely pathogenic, and 18 as variants of uncertain significance (Supplemental Table 2).

**Fig. 3:**
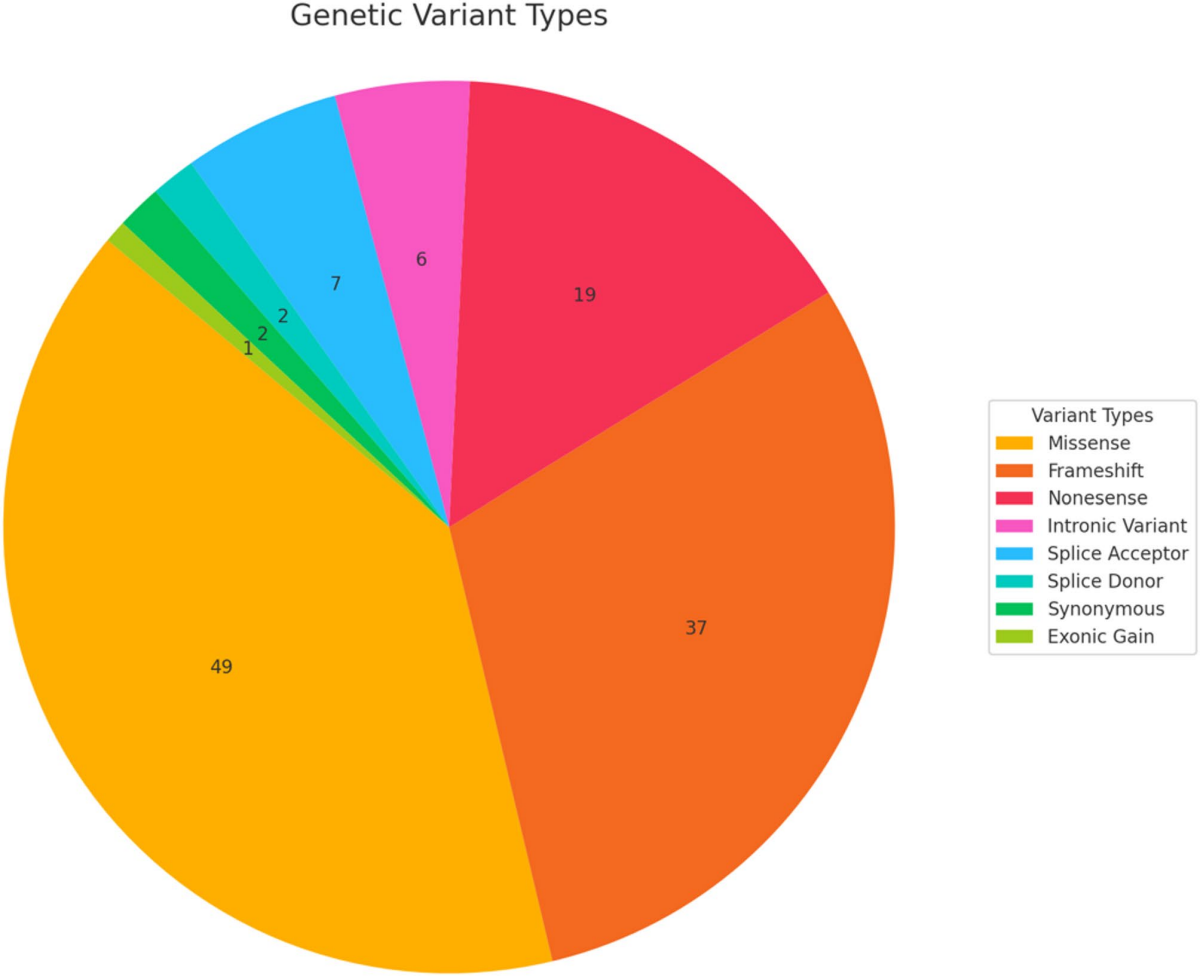
Frequency of allele variant’s molecular consequences in patient population. Molecular consequence frequencies are shown, with variants causing a missense being the most common, followed by variants causing a frameshift

Across all variants, the most common genes were USH2A (80), MYO7A [17], and ADGRV1 [11]. Pathogenic variants were found in 7 genes: *ADGRV1*, *CDH23*, *CLRN1*, *MYO7A*, *USH1C*, *USH2A*, and *WHRN*. The most common molecular consequences of each mutation were missense (53), followed by frameshift (38), and then nonsense mutations [17]. A detailed breakdown of these is shown in Fig. 3. Nine novel pathogenic or likely pathogenic variants were identified in four genes (ADGRV1, MYO7A, USHG1C, and USH2A) (Table 2).

**Table 2:** Novel variants identified on genetic testing

Gene	cDNA	Amino Acid	Molecular Consequence	Variant Classification
*ADGRV1*	c.18782T > C	p.Leu6261Ser	Missense	Pathogenic
*ADGRV1*	c.18782T > C	p.Leu6261Ser	Missense	Pathogenic
*MYO7A*	c.318 C > G	p.Asn106Lys	Missense	Likely Pathogenic
*MYO7A*	c.2659del	p.Lys887fs	Frameshift	Pathogenic
*MYO7A*	c.4920delC	p.Glu1642fs	Frameshift	Likely Pathogenic
*USH1C*	c.907 C > A	p.Arg303Ser	Missense	Pathogenic
*USH2A*	c.9995 C > A	p.Ser3332Ter	Nonsense	Pathogenic
*USH2A*	c.14406_14407insTC_c.14407_14420del	p.Ile4803fs	Frameshift	Pathogenic
*USH2A*	c.9003 A > G	p.Asn3001Lys	Missense	Pathogenic

## Discussion

USH is categorized clinically into three types (USH 1–3) based predominantly on the severity of symptoms and age of onset. Some patients who present with atypical audiovestibular or retinal findings do not fit into any of the three types and are clinically categorized as atypical USH. For each form of USH, the clinical manifestation can also vary according to the type and location of the causative mutation, ranging from nonsyndromic HL to isolated RP, further complicating the clinical picture [21, 22, 23]. This has become the method of choice for genetic studies of USH, as most of the USH-causing mutations are private, most of the involved genes are of a large size, and there are still new USH genes to be discovered.

As our understanding of the genetic basis of USH continues to expand, this system of Usher syndrome (USH) classification has become increasingly insufficient to encompass the current state of knowledge. With the advent of next generation sequencing (NGS) based screening strategies, the number of causative mutations identified for each USH associated gene has grown rapidly [21, 23]. This includes variants in common USH genes which result in atypical USH phenotypes, complicating the paradigm of assigning USH genes to either USH1, USH2, or USH3 [23, 24, 25]. Similarly, to better clarify genotype-phenotype relationships in *USH2A*, Molina-Ramirez et al. created an allelic hierarchy model of *USH2A* based on a cohort of isolated RP and *USH2A* patients with known genotypes for the purpose of predicting the phenotypic effects of specific categories of allelic variants [26]. This is even more crucial for American minorities, as they are generally under-represented in genetic testing studies due to their lower enrollment and consequently only limited published information is available on the main causative USH genes/variants in non-White Americans. This constitutes a critical gap in knowledge, which will presumably exclude minority populations from future precision medicine interventions for USH if left unaddressed [21].

We have reported here the genetic characterization of an ethnically diverse South Florida population with USH. Similarly to what has been reported in the past, the gene most commonly affected across nearly all of our race-ethnicity groups was *USH2A*, followed by *MYO7A* [8, 9]; our black or African American patient subset was comprised of one patient with heterozygous mutations in *MYO7A* and another with a single heterozygous mutation in *USH2A*. The diagnostic yield of our cohort (47/70; 67%) was low compared to several previous studies that reported a detection rate of about 80% [14, 26, 27, 28, 29]. Our enrichment for minorities may have produced results that are not representative of the general population. Furthermore, our analyses were based on self-identification in the electronic health record that may not be complete and accurate.

## Conclusions

This is the first genetic characterization of an ethnically diverse South Florida population with USH, which can help direct patient diagnosis and medical care. Genetic testing resolved two individuals with unknown subtype. Our data do not suggest a significant difference in the inclusion of molecular testing in patients of different races and ethnicities. We do, however, report a significant difference in positive diagnosis rates between White: Non-Hispanic or Latino patients and those of another race or ethnicity and between Black or African American patients and Unknown or not Reported: Non-Hispanic or Latino patients. The difference in positive diagnosis rates between Black or African American and White: Non-Hispanic or Latino patients was shown to approach statistical significance. Notably, our sample size is small and would benefit from the inclusion of a larger sample to compare diagnostic testing rates between groups. Furthermore, it should be noted that our data included a number of patients who had unknown or not reported racial/ethnic data. It is known that electronic medical records often reflect inaccurate patient data regarding race and ethnicity, which can impede efforts to address health care disparities between racial and ethnic groups [30]. Our results point to the need for accurate patient demographic data to identify disparities in health care access. As clinical trials for treatment commence, molecular testing all individuals is imperative.

## Electronic Supplementary Material

Below is the link to the electronic supplementary material.

Supplementary Material 1

## Data Availability

The datasets used and/or analyzed during the current study are available from the corresponding author on reasonable request.
