# Supplementary material for: Outcomes of genetic testing for Usher syndrome in a diverse population cohort from South Florida
Source: Hum Genomics. 2025 Jun 18;19:68. doi: 10.1186/s40246-025-00775-0 (PMC12177953; doi:10.1186/s40246-025-00775-0)
Supplement: Supplementary file 1 — Supplementary Material 1 [file 40246_2025_775_MOESM1_ESM.docx]

**Supplemental Table 1. All Pathogenic Variants by Race/Ethnicity and Affected Gene**

| **Race/Ethnicity** | **Gene 1** | **Variant DNA** | **Frequency** |
| --- | --- | --- | --- |
| American Indian or Alaska Native | *CEP78* | c.254-1G>T | 1 |
|  |  |  |  |
| Asian | *HARS1* | c.1361A>C | 2 |
|  |  |  |  |
|  | *MYO7A* | c.1903T>C | 2 |
|  |  |  |  |
|  | *USH2A* | c.5698T>G | 1 |
|  |  | c.4222C>T | 1 |
|  |  | c.9860_9873del | 1 |
|  |  |  |  |
| Black or African American | *MYO7A* | c.3665G>A | 1 |
|  |  | c.4972C>T | 1 |
|  |  |  |  |
|  | *USH2A* | c.2276G>T | 1 |
|  |  |  |  |
| Unknown or Not Reported:Non-Hispanic or Latino | *ADGRV1* | c.12775del | 1 |
|  |  | c.17668_17669del | 1 |
|  |  |  |  |
|  | *MYO7A* | c.318C>G | 1 |
|  |  | c.494C>T | 1 |
|  |  |  |  |
|  | *USH2A* | c.2081G>A | 1 |
|  |  | c.2299delG | 1 |
|  |  | c.5857+2T>C | 1 |
|  |  | c.8232del | 2 |
|  |  | c.920_923dup | 1 |
|  |  |  |  |
|  | *WHRN* | c.1573_1574del | 2 |
|  |  |  |  |
| Unknown or Not Reported:Unknown or Not Reported | *ADGRV1* | c.17668_17669del | 2 |
|  |  |  |  |
|  | *MYO7A* | c.1903T>C | 2 |
|  |  | c.2283-1G>T | 1 |
|  |  | c.4920delC | 1 |
|  |  |  |  |
|  | *USH2A* | c.11864G>A | 2 |
|  |  | c.12067-2A>G | 1 |
|  |  | c.12575G>A | 2 |
|  |  | c.1680or1678delC | 1 |
|  |  | c.2299delG | 3 |
|  |  | c.3368A>G | 2 |
|  |  | c.7595-3C>G | 1 |
|  |  | c.8522G>A | 1 |
|  |  |  |  |
| White:Hispanic or Latino | *ADGRV1* | c.12775del | 1 |
|  |  | c.17020-?_17856+?du | 1 |
|  |  | c.17668_17669del | 1 |
|  |  | c.18782T>C | 2 |
|  |  | c.5953A>C | 2 |
|  |  | c.9447G>A | 2 |
|  |  | c.9877C>T | 1 |
|  |  |  |  |
|  | *CLRN1* | -71U G>A | 1 |
|  |  |  |  |
|  | *MYO7A* | c.2283-1G>T | 1 |
|  |  | c.3962A>G | 1 |
|  |  | c.-46-1G>T | 1 |
|  |  | c.5510T>A | 1 |
|  |  | c.635G>A | 2 |
|  |  |  |  |
|  | *USH1C* | c.907C>A | 1 |
|  |  |  |  |
|  | *USH2A* | c.10712C>T | 2 |
|  |  | c.12067-2A>G | 1 |
|  |  | c.12575G>A | 1 |
|  |  | c.13374delA | 1 |
|  |  | c.13436T>G | 1 |
|  |  | c.14180G>A | 1 |
|  |  | c.14248C>T | 1 |
|  |  | c.14406_14407insTC_c.14407_14420del | 1 |
|  |  | c.14408_14420del | 1 |
|  |  | c.1992dup | 1 |
|  |  | c.2276G>T | 1 |
|  |  | c.2299delG | 7 |
|  |  | c.8917_8918del | 1 |
|  |  | c.956G>A | 1 |
|  |  |  |  |
| White:Non-Hispanic or Latino | *CDH23* | c.1515-12G>A | 1 |
|  |  | c.3598G>T | 1 |
|  |  |  |  |
|  | *CLRN1* | c.144T>G | 4 |
|  |  |  |  |
|  | *MYO7A* | c.1190C>A | 1 |
|  |  | c.2659del | 1 |
|  |  | c.3719G>A | 1 |
|  |  | c.6025delG | 1 |
|  |  |  |  |
|  | *USH1C* | c.216G>A | 1 |
|  |  | c.238dupC | 1 |
|  |  | c.496+1G>T | 1 |
|  |  | c.496+3G>C | 1 |
|  |  |  |  |
|  | *USH2A* | c.10073G>A | 1 |
|  |  | c.1055C>T | 1 |
|  |  | c.11156G>A | 1 |
|  |  | c.11864G>A | 1 |
|  |  | c.12152_12153insTT | 1 |
|  |  | c.12575G>A | 3 |
|  |  | c.13392G>A | 1 |
|  |  | c.1478A>G | 1 |
|  |  | c.1481A>G | 1 |
|  |  | c.1972-1G>A | 1 |
|  |  | c.2276G>T | 3 |
|  |  | c.2299delG | 5 |
|  |  | c.2301del | 1 |
|  |  | c.2670del | 1 |
|  |  | c.3187_3188delCA | 1 |
|  |  | c.3368A>G | 1 |
|  |  | c.4222C>T | 1 |
|  |  | c.653T>A | 1 |
|  |  | c.6937G>T | 1 |
|  |  | c.7595-2144A>G | 1 |
|  |  | c.802G>A | 2 |
|  |  | c.8682-9A>G | 1 |
|  |  | c.8834G>A | 1 |
|  |  | c.9003A>G | 1 |
|  |  | c.920_923dup | 1 |
|  |  | c.9459C>A | 1 |
|  |  | c.9676C>T | 1 |
|  |  | c.9860_9873del | 1 |
|  |  | c.9995C>A | 1 |
|  |  |  |  |
| White:Unknown or Not Reported | *USH2A* | c.2299delG | 1 |
|  |  | c.577G>T | 1 |
